# Personal barriers to addressing intimate partner abuse: a qualitative meta-synthesis of healthcare practitioners’ experiences

**DOI:** 10.1186/s12913-021-06582-2

**Published:** 2021-06-09

**Authors:** Laura Tarzia, Jacqui Cameron, Jotara Watson, Renee Fiolet, Surriya Baloch, Rebecca Robertson, Minerva Kyei-Onanjiri, Gemma McKibbin, Kelsey Hegarty

**Affiliations:** 1 Department of General Practice, The University of Melbourne, Melbourne, Victoria Australia; 2 Centre for Family Violence Prevention, The Royal Women Hospital, Parkville, Victoria Australia; 3 School of Health and Society, University of Wollongong, Wollongong, New South Wales Australia; 4 School of Nursing & Midwifery, Deakin University, Geelong, Victoria Australia; 5 Department of Social Work, The University of Melbourne, Melbourne, Victoria Australia

**Keywords:** Intimate partner violence, Health practitioners, Qualitative meta-synthesis, Barriers

## Abstract

**Background:**

Healthcare practitioners (HCPs) play a crucial role in recognising, responding to, and supporting female patients experiencing intimate partner abuse (IPA). However, research consistently identifies barriers they perceive prevent them from doing this work effectively. These barriers can be system-based (e.g. lack of time or training) or personal/individual. This review of qualitative evidence aims to synthesise the personal barriers that impact HCPs’ responses to IPA.

**Methods:**

Five databases were searched in March 2020. Studies needed to utilise qualitative methods for both data collection and analysis and be published between 2010 and 2020 in order to qualify for inclusion; however, we considered any type of healthcare setting in any country. Article screening, data extraction and methodological appraisal using a modified version of the Critical Appraisal Skills Program checklist for qualitative studies were undertaken by at least two independent reviewers. Data analysis drew on Thomas and Harden’s thematic synthesis approach.

**Results:**

Twenty-nine studies conducted in 20 countries informed the final review. A variety of HCPs and settings were represented. Three themes were developed that describe the personal barriers experienced by HCPs: *I can’t interfere* (which describes the belief that IPA is a “private matter” and HCPs’ fears of causing harm by intervening)*; I don’t have control* (highlighting HCPs’ frustration when women do not follow their advice)*; and I won’t take responsibility* (which illuminates beliefs that addressing IPA should be someone else’s job)*.*

**Conclusion:**

This review highlights the need for training to address personal issues in addition to structural or organisational barriers. Education and training for HCPs needs to: encourage reflection on their own values to reinforce their commitment to addressing IPA; teach HCPs to relinquish the need to control outcomes so that they can adopt an advocacy approach; and support HCPs’ trust in the critical role they can play in responding. Future research should explore effective ways to do this within the context of complex healthcare organisations.

**Supplementary Information:**

The online version contains supplementary material available at 10.1186/s12913-021-06582-2.

## Background

Intimate partner abuse (IPA) is a global epidemic which disproportionately affects women and their children. It is consistently associated with a range of serious negative physical, mental and reproductive health outcomes and is a major cause of injury, morbidity and mortality in women [1, 2]. Defined as behaviour perpetrated by a current or former intimate partner that causes physical, psychological, financial or sexual harm [3], around one in every three women worldwide has experienced IPA in their lifetime [4]. The health impacts of IPA can linger long after a woman has left an abusive relationship, leading to many chronic conditions such as depression, post-traumatic stress, menopausal issues, sexually-transmitted infections and diabetes [2]. The corresponding economic and social costs of IPA are enormous [5].

IPA is a complex, “wicked” [6] problem, meaning that it requires an inter-disciplinary and multisectoral response in order to address it effectively. In line with this, research and policy have increasingly recognised that health systems form a critical part of this response [7]. Women who are experiencing IPA make more frequent use of healthcare services than women without a history of violence [8, 9]. This is the case across all levels of the health system, including primary care, hospital emergency departments, sexual and reproductive health services and mental health services [8]. Studies suggest that women are comfortable disclosing IPA to a HCP providing they feel safe and free from judgement [7, 10]. Consequently, healthcare practitioners (HCPs) are well-placed to identify, respond and provide supportive care [7]. The World Health Organization strongly recommends that HCPs incorporate a response to IPA into their daily practice and has developed guidelines around how to do this effectively and sensitively [11].

At the same time, identifying and responding to IPA is not easy work. Research has consistently identified numerous barriers that prevent HCPs from addressing IPA in their daily practice [12–14]. Sprague and colleagues, in a 2010 systematic review of 22 qualitative studies [12], identified lack of time, personal discomfort with the topic of IPA and lack of knowledge as the primary barriers preventing HCPs from undertaking routine screening. More recently, Saletti-Cuesta and colleagues [13] conducted a systematic review of 46 qualitative studies and synthesised the opinions and perceptions of HCPs regarding IPA. In their section on barriers, they identified organizational issues such as lack of time and knowledge, but also flagged over-reliance on the biomedical model and personal issues around managing emotions when dealing with IPA. Despite the existence of clinical guidelines [11], and the development of promising training programs [15, 16], many of these barriers persist. This is problematic given the harmful impacts on women’s wellbeing when their expectations for care are not met by HCPs [17].

**Fig. 1:**
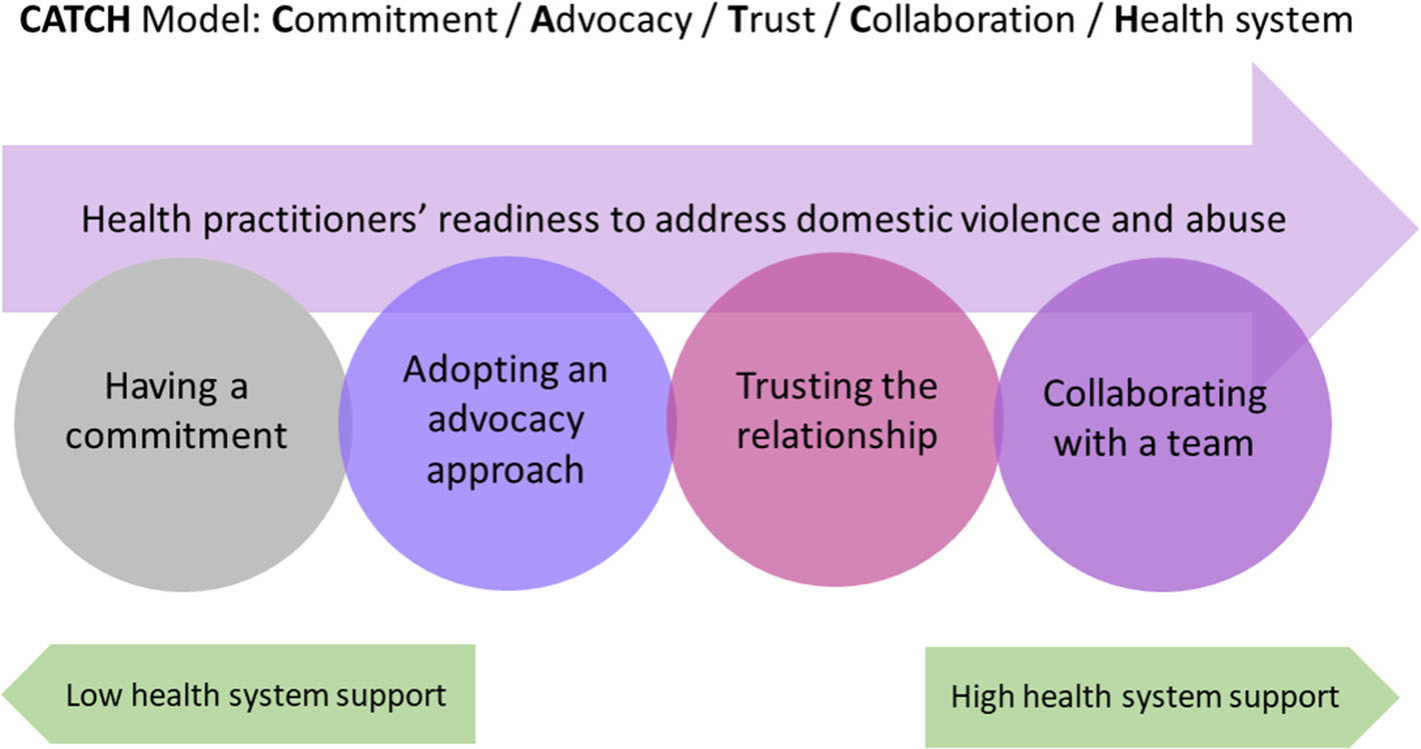
CATCH Model – Factors influencing practitioner readiness to respond to IPA [21]

In order for behaviours to become normalised into practice, particular criteria need to be met across a number of levels [18, 19], both personal and structural. In the context of responding to IPA, these criteria include HCPs understanding *how* and *why* they need to be doing this work, as well as having clarity around *whose responsibility* it should be [19, 20]. Indeed, a recent meta-synthesis of qualitative studies on the factors promoting HCPs’ readiness to respond to IPA [21] suggested that having a commitment, adopting an advocacy approach, and trusting the relationship in the health setting were three out of the five key elements. When HCPs had personal values that supported a commitment to addressing IPA, were willing to engage in a woman-centred way and felt confident in their ability to support women in the context of their role, they were able to engage successfully in identifying and responding to IPA (see Fig. 1). These are clearly factors that relate to the individual HCP rather than relating to the organisation or the broader social context. The majority of the literature relating to barriers, however, focuses on structural or organizational issues [12, 13].

This qualitative meta-synthesis addresses this gap by comprehensively reviewing the evidence relating to *personal* barriers experienced by HCPs that prevent them from responding effectively to women patients exposed to IPA. We chose to focus on qualitative evidence in order to understand the subjective experiences, perceptions and beliefs of HCPs. Whilst previous reviews have included some elements relating to personal factors, to date no studies have solely focused on understanding barriers at the level of the individual practitioner. We concentrated on studies published since 2010, when Sprague and colleagues published their review, so that the latest evidence on the issue would be captured. We were guided by the research question: *What are the personal barriers that health practitioners perceive prevent them from addressing intimate partner abuse against women?*

## Methods

### Search strategy

The protocol for this review was registered with PROSPERO (CRD4202019645). Five databases were searched in March 2020: EMBASE, Medline and PsycINFO through the OVID platform and SocIndex and CINAHL through the EBSCO platform). These searches were supported by reference checking of included studies, forward citations and consultation with field experts. The search used subject headings, text words and keywords for: healthcare professional, intimate partner abuse and qualitative research. An example of the OVID search is provided in Additional file 1.

#### Inclusion criteria

We included primary studies published between 2010 and 2020 with both qualitative methods for data collection (e.g. focus groups, interviews) and data analysis (e.g. thematic analysis, grounded theory). The main focus of included studies needed to be on HCP-perceived personal barriers to addressing IPA against women; studies with minimal data on this topic were excluded. The study population needed to include HCPs, with HCP data analysed separately from other participants (e.g. service users). Randomised controlled trials, cross-sectional studies, clinical case studies, cohort studies, case-control studies, and review articles were excluded.

### Selection of studies

The web-based application Covidence [22] was used to manage references during the review period. Titles and abstracts were imported into Covidence and independently screened by two reviewers (JW, RR). Duplicates were removed throughout the review process. Studies determined to be potentially relevant or whose eligibility was uncertain were retrieved for full-text review. The two reviewers then independently assessed the full-text articles for the remaining studies to ascertain eligibility for inclusion. A third reviewer voted for inclusion or exclusion if there was any disagreement in the screening process. The list of included studies was further reviewed by the first author to finalise the data set.

### Data extraction & analysis

Data and supporting information were extracted into a template developed for this review. Supporting information for each study included setting and participant information, study design, ethical issues and data analysis. Data were defined as primary study author interpretations of their findings, with supporting quotations from participants, as well as the overall conclusions of the study. These were extracted from the “Results” or “Findings” sections of the included studies, as well as from the “Discussion” and “Conclusion” sections where necessary. The extraction templates were imported into the software program, QSR NVivo [23] for analysis.

A thematic synthesis was completed following Thomas and Harden’s approach [24]. This involved immersion in the data, line-by-line coding, organisation of codes into themes (including analytical themes), and interpretation to develop further concepts and understanding [24]. Four members of the review team (LT, JC, JW and RF) were involved in coding the included studies using this process. The codes were checked several times with other team members to ensure they were an accurate representation of the data.

### Methodological quality

The methodological quality of included studies was assessed using the Critical Appraisal Skills Programme (CASP) checklist for qualitative studies [25]. This checklist assists in assessing a study’s quality across several domains (e.g. data collection and analysis, ethical considerations, recruitment). The CASP does not have a scoring system. Instead, it asks the user to indicate whether individual studies address each of the criteria (yes/no/partially/unclear). Based on the CASP criteria, as well as an assessment of any other methodological or ethical issues not covered by the CASP, we gave each study a rating of ‘no or very minor concerns’, ‘minor concerns’, ‘moderate concerns’ or ‘serious concerns’. This rating system follows the GRADE CerQUAL method for assessing methodological quality [26]. Two reviewers (JC, SB) independently evaluated all included studies using this process, with a third reviewer consulted in the event of discrepancies.

## Results

**Fig. 2:**
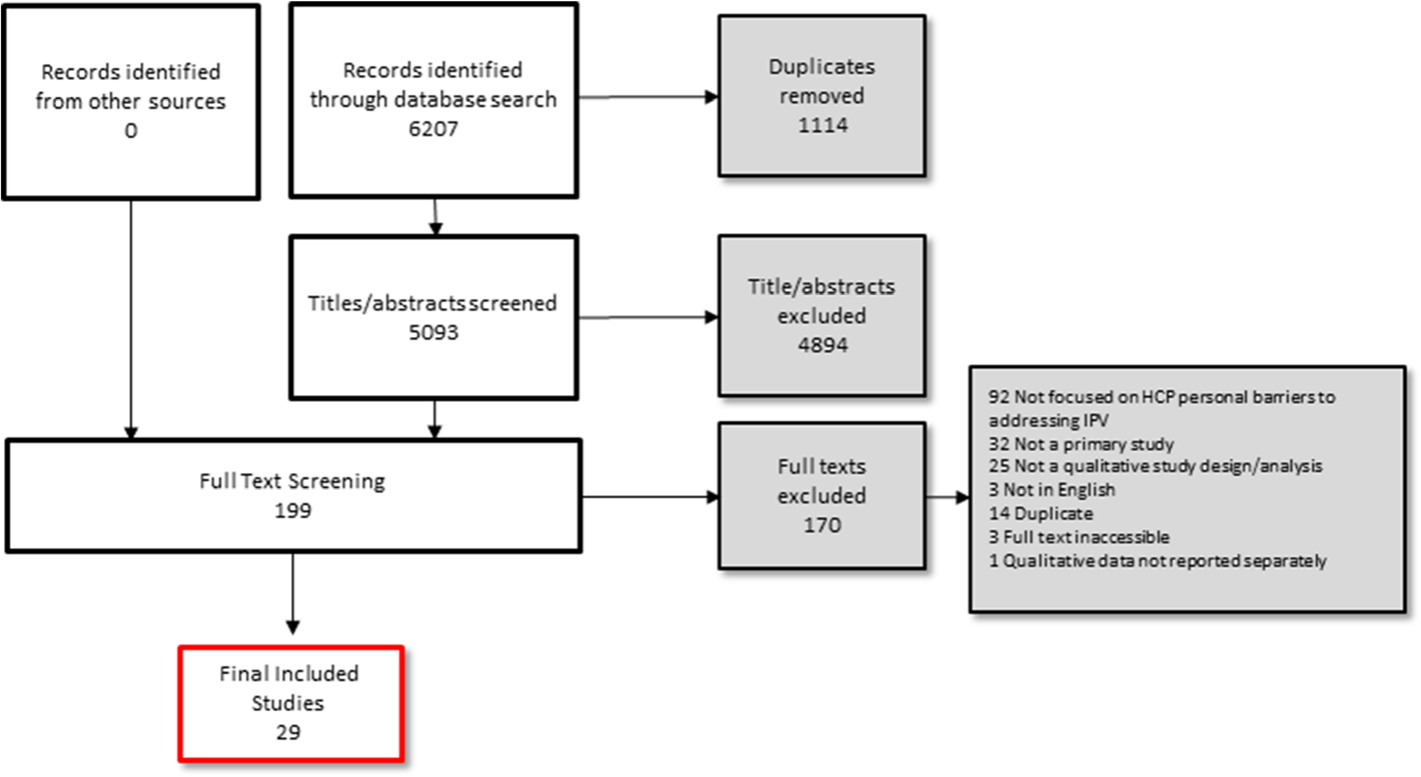
Flow of Studies

We identified 29 studies published between 2010 and 2020. Figure 2 depicts the flow of studies presented following the PRISMA (Preferred Reporting Items for Systematic reviews and Meta-Analyses) guidelines [27].

Included studies represented 20 countries. Studies were conducted in a range of health care settings, including hospitals, health care and primary health care clinics. Health care professionals included nurses (advanced practice nurses, primary health care nurses and district nurses), physicians/doctors (including general practitioners), midwives, licensed and unlicensed primary health care providers and mental health therapists. The methods used for data collection consisted primarily of interviews or focus groups. A summary of the characteristic of the included studies is provided in Table 1.

**Table 1:** Characteristics of included studies

Study Number	Authors	Year (Country)	Objective Summary	HCP Setting	Sample Size	Data Collection / Analysis Method	Years of clinical experience
1	Aziz & El-Gazzar [28]	2019 (Egypt)	To explore the attitude of HCPs about screening for and dealing with IPA in the health care setting and to assess the physicians’ screening behaviour.	Hospital	*N* = 22	Focus groups / Thematic analysis	Average: < 5 years
2	Baig et al. [29]	2012 (Colombia)	To examine provider barriers and facilitators to screening for IPA	Hospital	*N* = 27	Interviews / Un-named analysis method describing content analysis	Not provided
3	Colarossi et al. [30]	2010 (USA)	To expand current knowledge by comparing licensed family planning service providers (advanced practice clinicians and social workers) and unlicensed ones (health care assistants) who work in a setting guided by institutional policy and procedure for IPA screening.	Family Planning Centre	*N* = 64	Focus Groups / Grounded Theory	Range: < 5 years - over 10 years
4	Conn et al. [31]	2014 (Canada)	To explore orthopedic surgery residents’ knowledge of IPA and their preparedness to screen patients for IPA in a fracture clinic setting with a view to developing targeted IPA education and training.	Hospital	*N* = 64	Focus Groups / Unspecified inductive analysis	Range: 1–5 years
5	Columbini et al. [32]	2013 (Malaysia)	To analyse barriers and opportunities to implement and integrate effective health service responses to IPA at different levels of the health system.	Hospital	*N* = 54	Interviews / Framework Analysis	Not provided
6	Djikanovic et al. [33]	2010 (Serbia)	To identify HCPs’ perceptions and attitudes regarding IPV in Serbia (Belgrade), as well as how they perceive barriers for providing appropriate help for women who have experienced IPA.	Healthcare Clinic	*N* = 71	Focus Groups / Qualitative content analysis	Not provided
7	Efe & Taskin [34]	2012 (Turkey)	To delineate the factors that prevent the adequate provision of nursing services to women subjected to IPA.	Hospital	*N* = 30	Interviews / Descriptive analysis	Not provided
8	Finnbogadottir & Dykes [35]	2012 (Sweden)	To explore midwives’ awareness of and clinical experience regarding IPA among pregnant women in southern Sweden.	Hospital	*N* = 16	Focus Groups / Content text analysis	Range: 4–36 years
9	Gallagher [36]	2014 (UK)	To explore how educational psychologists conceptualised IPA and the role they could have in working with schools and children and families.	Urban local services	N = 5	Interviews / Thematic analysis	Range: 4–15 years
10	Guruge [37]	2012 (Sri Lanka)	To explore how Sri Lankan nurses perceive their role in caring for women experiencing IPA.	Hospital	N = 30	Interviews / Thematic analysis	Range: 1–15 years
11	Husso et al. [38]	2012 (Finland)	To explore how HCPs make sense of IPA interventions and the organisational practices of these interventions.	Health Clinic	N = 30	Focus Groups / Framework analysis	Not provided
12	Mauri et al. [39]	2015 (Italy)	To explore midwives’ knowledge and clinical experience of IPA among pregnant women, with particular emphasis on their perceptions of their professional role.	Hospital & local health	*N* = 15	Interviews / Content analysis	Range: 8 months to 35 years
13	McCauley et al. [40]	2017 (Pakistan)	To investigate the knowledge and perceptions of IPA among doctors who provide routine antenatal and postnatal care at healthcare facilities in Pakistan.	Hospital	*N* = 25	Interviews/ Thematic framework analysis	Range: 2–10 years
14	Papadakaki et al. [41]	2014 (Greece)	To explore the perceptions and practices of general practitioners (GPs) regarding the identification and management of IPA in primary care settings.	Primary Care	*N* = 18	Focus Groups / Thematic analysis	Mean 12 years
15	Pau [42]	2015 (Malaysia)	To examine factors that influence Malaysian health care providers’ attitudes, knowledge, and responses to IPA survivors, including their perceptions of IPA, factors that influenced the ways they work with IPV survivors, factors they perceived influenced IPA survivors’ help-seeking behaviors, and their recommendations for improving IPA training.	Hospital, NGOs and department of social welfare	*N* = 17	Interviews / Constant comparative analysis	Range 1–30 years (md = 5 years)
16	Pitter [43]	2016 (Jamaica)	To improve the capacity of midwives to identify and treat pregnant women experiencing IPA in Jamaica.	Hospital	N = 6	Focus Groups / content analysis	Range: < 1–11 years
17	Rahmqvist et al. [44]	2019 (Sweden)	To describe emergency nurses’ experiences when caring for victims of violence and their family members in emergency departments.	Hospital	*N* = 12	Interviews / Qualitative content analysis	Median 4.5 years
18	Robinson [45]	2010 (USA)	To identify how registered nurses screen for intimate partner violence in the emergency department.	Hospital	*N* = 13	Interviews / Colaizzi’s seven step analysis	Not provided
19	Rose et al. [46]	2011 (UK)	To explore the facilitators and barriers to disclosure of IPA from a service user and professional perspective.	Mental Health Services	*N* = 20	Interviews / Unspecified thematic analysis	Range: 4–29 years
20	Sormanti & Smith [47]	2010 (USA)	To explore health physicians’ reactions and ideas about IPA screening in the emergency department setting.	Hospital	N = 25	Focus Groups / Content analysis	Not provided
21	Spangaro et al. [20]	2011 (Australia)	To understand challenges, and enablers of screening apply this to a model of how health policies become routinized in practice.	Health Services	*N* = 59	Focus Groups / Unspecified inductive analysis	Not provided
22	Sun et al. [48]	2019 (Hong Kong)	To investigate the barriers of Hong Kong primary care physicians toward managing IPA, including barriers of recognition, management, and referrals of these patients.	Hospital	*N* = 26	Focus Groups / Content analysis	Not provided
23	Sundborg et al. [49]	2017 (Sweden)	To improve understanding of district nurses’ experiences of encountering women exposed to IPA.	Primary Care	*N* = 11	Interviews / content analysis	Not provided
24	Usta et al. [50]	2014 (Lebanon)	To explore physicians’ attitudes about responding to IPA, their perception of the physician’s role, and the factors that influence their response.	Primary Care	*N* = 67	Interviews / Thematic analysis	Mean 19 years
25	Van der Wath [51]	2019 (South Africa)	To uncover discourses that may help understand emergency nurses’ responses towards women exposed to IPA.	Hospital	N = 15	Focus Groups / Unspecified thematic analysis	Not provided
26	Visentin et al. [52]	2015 (Brazil)	To identify the actions conducted by primary health care nurses for women in situations of IPA.	Health Units	N = 17	Interviews / Content analysis	Range: < 1–21 years
27	Watson et al. [53]	2017 (UK)	To address the gap in the literature concerning the key conditions therapists experience when working with women over the age of 45 presenting with IPA.	Mental Health	*N* = 17	Interviews / Grounded theory approach	Range 1–20 years
28	Zakar et al. [54]	2011 (Pakistan)	To investigate the response of primary health care physicians in diagnosing and treating the victims of IPA in Pakistan.	Hospital	*N* = 24	Interviews / Unspecified thematic analysis	Range 3–26 years
29	Zijlstra et al. [55]	2017 (Netherlands)	To examine factors facilitating and constraining the identification and management of IPA at an emergency department.	Hospital	N = 18	Interviews / content analysis	Range: < 1–15 years

### Quality of included studies

Using the CASP [25], we determined that 21 [20, 28–30, 32, 33, 35, 37, 39–42, 44–46, 49, 51–54, 56] of the studies were methodologically sound and had ‘no or very minor concerns’. In seven studies [31, 34, 36, 48, 50, 51, 55] only minor concerns were found. One study [43] had moderate concerns. Overall, studies demonstrated minor issues with recruitment, lack of detail around data collection and analysis, and missing information about ethics approval. The results of our assessment are shown in Table 2 below.

**Table 2:** CASP Assessment Results

Author (year)	Statement of aim?	Qualitative methodology appropriate?	Research design appropriate?	Recruitment strategy appropriate?	Relationship between researcher & participants adequately considered?	Ethical issues taken into consideration?	Data analysis sufficiently rigorous?	Findings supported by evidence?	Other limitations?	Overall assessment of quality
Aziz & El-Gazzar [28]	Yes	Yes	Yes	Yes	Yes	Yes	Yes	Yes	N/A	No or very minor concerns
Baig et al. [29]	Yes	Yes	Yes	Yes	Partial	Yes	Yes	Yes	N/A	No or very minor concerns
Colarossi et al. [30]	Yes	Yes	Yes	Yes	Yes	Yes	Yes	Yes	N/A	No or very minor concerns
Conn et al.	Yes	Yes	Yes	Partial	Yes	Yes	Yes	Yes	Participants were mostly junior residents with less experience.	Minor concerns
Columbini et al. [32]	Yes	Yes	Yes	Unclear	Yes	Yes	Yes	Yes	N/A	No or very minor concerns
Djikanovic et al. [33]	Yes	Yes	Yes	Yes	Unclear	Yes	Yes	Yes	Methods lack detail.	No or very minor concerns
Efe & Taskin [34]	Yes	Yes	Yes	yes	Unclear	Partial	Yes	Yes	No details on ethics and data analysis.	Minor concern.
Finnbogadottir & Dykes [35]	Yes	Yes	Yes	Yes	Yes	Yes	Yes	Yes	N/A	No or very minor concerns
Gallagher [36]	Yes	Yes	Yes	Partial	Unclear	Partial	Yes	Yes	Concerns around recruitment and ethics.	Minor concerns
Guruge [37]	Yes	Yes	Yes	Yes	Yes	Yes	Yes	Yes	N/A	No or very minor concerns
Husso et al. [38]	Yes	Yes	Yes	Yes	Yes	Yes	Yes	Yes	N/A	No or very minor concerns
Mauri et al. [39]	Yes	Yes	Yes	Yes	Partial	Yes	Yes	Yes	No limitations acknowledged	No or very minor concerns
McCauley et al. [40]	Yes	Yes	Yes	Yes	Yes	Yes	Yes	Yes	N/A	No or very minor concerns
Papadakaki et al. [41]	Yes	Yes	Yes	Yes	Yes	Yes	Yes	Yes	N/A	No or very minor concerns
Pau [42]	Yes	Yes	Yes	Yes	Yes	Yes	Yes	Yes	N/A	No or very minor concerns
Pitter [43]	Yes	yes	Yes	Partial	yes	Yes	Partial	Partial	Concerns around sample recruitment, data analysis and discussion.	Moderate concerns
Rahmqvist et al. [44]	Yes	yes	yes	yes	yes	yes	yes	yes	N/A	No or very minor concerns
Robinson [45]	Yes	Yes	Yes	Yes	Yes	Yes	Yes	Yes	N/A	No or very minor concerns
Rose et al. [46]	Yes	Yes	Yes	Yes	Yes	Yes	Yes	Yes	N/A	No or very minor concerns
Sormanti & Smith [47]	Yes	Yes	Yes	Yes	Yes	Partial	Yes	Yes	Concerns about informed consents.	Minor concerns
Spangaro et al. [20]	Yes	Yes	Yes	Yes	Yes	Yes	Yes	Yes	N/A	No or very minor concerns
Sun et al. [48]	Yes	Yes	Yes	Yes	Partial	Partial	Partial	Yes	Concern around ethical issues and data collection.	Minor concerns
Sundborg et al. [49]	Yes	Yes	Yes	Yes	Yes	Yes	Yes	Yes	N/A	No or very minor concerns
Usta et al. [50]	Yes	Yes	Yes	Yes	Partial	Yes	Yes	Yes	Concerns about data collection (short interviews)	Minor concerns
Van der Wath [51]	Yes	Yes	Yes	Partial	Partial	Yes	Yes	Yes	N/A	No or minor concerns
Visentin et al. [52]	Yes	Yes	Yes	Yes	Yes	Yes	Yes	Yes	N/A	No or very minor concerns
Watson et al. [53]	Yes	Yes	Yes	Yes	Yes	Yes	Yes	Yes	N/A	No or very minor concerns
Zakar et al. [54]	Yes	Yes	yes	Yes	Yes	Yes	Yes	Yes	N/A	No or very minor concerns
Zijlstra et al. [55]	Yes	Yes	Yes	Yes	Yes	Partial	Yes	Yes	Lack of detail on ethical approval	Minor concerns

### Key themes

Thematic synthesis of the included studies led to the development of three key themes that describe the personal barriers HCPs perceived prevented them from responding effectively to IPA. These themes were: *I can’t interfere*; *I don’t have control* and; *I won’t take responsibility.* Each theme is described below with supporting quotations.

### I can’t interfere

A strong theme common to 20 of the articles [29, 33–35, 37, 39–47, 49–51, 54, 55] was that even when IPA is suspected, the HCP cannot (or should not) interfere. The idea that IPA is a “private matter” was frequently mentioned as justification for this belief, particularly in communities where the institution of the family is valued above individual autonomy. For example, a Turkish study by Efe and Taskin [34] revealed that some nurses believed that problems within the family should remain within the family.

*We do not really try to find out family details, get to know what the structure is, how it happened, who did this to you or things like that. (p.447)*

Similarly, in Lebanon, Usta and colleagues [50] found that HCPs believed that they have “no right to intervene in such problems at all unless the patient or some family members asked them to interfere” (p.e315).

For some practitioners, the main reason for not interfering was being uncertain whether an enquiry or intervention may cause more harm than good for a woman experiencing IPA. A physician in a Hong Kong-based study by Sun and colleagues [48] commented:

*I don’t want to deepen the tension between the couple. If I take sides or agree with the wife, I might have made an early judgment. This might be harmful to their relationship. (p.7)*

In particular, HCPs feared that asking women about IPA would be perceived as offensive, as though they were making a judgement or assumption, potentially causing embarrassment or shame if their suspicion was incorrect.

*You might feel that between partners […] there is something that's not right. But, again, you cannot always look into it and ask… because you could also have misunderstood the situation, right?* [39]*(p.500)*

*I don’t know at what point you turn around and say “have you been a victim of domestic violence?” I think it has the potential to scare some people off* [46]*.(p.192)*

Connected to the perception that asking about IPA is offensive, HCPs were concerned about damaging their relationship with the patient if they enquired about IPA. One midwife in a Swedish study by Finnbogadöttir et al. commented:

*We are so terrifically concerned about our relationship, we midwives, so we don't dare bring matters to a head, because what if they don't like us and they switch midwives, then one is really worthless (said with emphasis)* [35]. *(p.194)*

HCPs also worried about the personal cost of addressing IPA with patients. Participants in two studies expressed reluctance to ask patients about IPA because they found it too emotionally distressing [44, 49]. For others, the risk related to potential legal repercussions stopped them [47]. On the other hand, in some countries (predominantly low-and-middle-income) the HCPs were fearful for their personal safety from the perpetrator [33, 35, 37, 40, 41, 43, 50]. For example, a HCP working in Lebanon commented that:

*No matter how much we want to help…we are absolutely unprotected on the street, in cafes, everyone carries a weapon. You do not know who will react* *[*33*]. (p.91)*

This theme highlights HCPs’ fear and reluctance to “get involved” in addressing IPA. For some HCPs in low-and-middle-income countries, there are legitimate concerns for personal safety, although it is possible that these fears stem from the assumption that they might be required to help a woman to leave an abusive relationship or confront the perpetrator. However; the literature suggests that there are many practical yet unobtrusive ways that a HCP can support women experiencing IPA that do not involve leaving the relationship [17] and need not endanger themselves or the patient. For other HCPs, not wanting to interfere appears to be related to the mistaken belief that women will be upset or offended if asked about IPA, and that this perceived harm outweighs the potential benefits.

### I don’t have control

This theme highlights HCPs’ feelings of frustration, resentment, helplessness and inadequacy when women experiencing IPA chose not to follow their instructions and advice. A particular focus for this frustration was a woman’s decision to remain with, or return to, an abusive partner. Ten of the studies included in the review discussed the exasperation felt by HCPs when their female patients did this [20, 30, 32, 33, 35, 41, 45, 52, 53, 55].

*The biggest obstacle in doing this work is one's own feelings and cynicism and that frustration. And somehow because we have no means for enforced commitment and care, so there she goes, back to being beaten again. I can't do anything* *[*38*]. (p.352)*

In one study in an emergency department in the Netherlands [55], a HCP described how they had grown so frustrated that they had “given up” responding to a woman who repeatedly presented with injuries relating to IPA:

*There is a known recidivist with proven family violence who comes to our ED twice a week. We offered a lot to her, but she keeps coming back and I think: “Oh, it’s her again.” Now I’ve given up (. . .) and stopped paying attention to the violence. (p.1054)*

The perception of a woman experiencing IPA as a “recidivist” is obviously problematic and demonstrates a lack of understanding about the dynamics of IPA. It suggests that – for some HCPs – the need to control the encounter stems from a lack of knowledge about women’s autonomy when in a controlling relationship.

Practitioners also experienced aggravation and disappointment when women chose not to follow advice they had provided about other matters (for example, contacting services). Some HCPs expressed regret that they could not force women to comply with what they felt was the best course of action.

*I offer [to set them up with] social work services, but when they refuse, I just want to shake them, because I can't help them* [30]. *(p.240)*

*It is the impotence of the professionals. You want to act in a faster way, you want to make her denounce and she does not want it* [52]*. (p540)*

Across eight studies [30, 32, 34, 35, 38, 47, 51, 53], HCPs described a degree of futility inherent in the work of identifying and responding to IPA. This disillusionment could then become a barrier to their future commitment to addressing this issue in their practice.

*I think that there are times when we subconsciously or consciously decide not to [screen]. You know that so many people, even if you identify it, are not going to do anything* [20]*. (p.134)*

*If you are a therapist, you want to fix someone. If somebody talks about something that you have no idea about, it makes you feel helpless because you feel like you can't do anything to help them* *[*53*]. (p.227)*

Many HCPs genuinely wanted to help their patients. Perceiving that their efforts were ineffective could lead to feelings of being overwhelmed and depressed. In extreme cases, this could lead to practitioner burn-out.

*What I am doing now, I feel it is not enough because I'm just doing the basic counselling, identify their problem and referring them to the other units, you see...most of them won't come back, but I feel very depressed because I can't do much* [32]. *(p.6)*

*I used to go home and be in tears worried that people were at risk and I should do more and I used to go out of my way, I’d be going in on my days off* [53]*(p.228)*

### I won’t take responsibility

Practitioners in 14 of the included studies [28, 29, 31, 32, 37, 38, 40, 41, 44–47, 49, 50, 54] described reluctance to make addressing IPA their responsibility. Major contributors to this reluctance were the perception of IPA as a “social problem” and the belief that the needs of women experiencing IPA are inherently complex and time-consuming.

Rahmqvist and colleagues [44] described how HCPs sometimes avoid talking with patients about IPA due to the fact that “it was considered difficult, requiring sensitivity and knowledge, and simply taking more time than participants felt they could give” (p.4). Other studies variously described HCPs being worried about opening a “can of worms” [48] or “Pandora’s box” [20, 55] by asking women about IPA. Related to this, many HCPs emphasized that – due to the perceived complexity of women’s needs – they felt unqualified to respond and felt it best not to enquire at all, preferring to make it someone else’s responsibility. A first-year resident working in a large hospital explained this succinctly in the below excerpt from a study by Sormanti and Smith [47]:

*…it’s [IPA] beyond the scope of our practice and there are others who are better qualified to handle the situations once they are discovered. (p.31)*

In Husso’s study in Finland [38], HCPs perceived that dealing with a patient’s emotional reactions would detract from their provision of medical care, and consequently was best left to others.

*What if it triggers it right there, where you need to assess the patient’s condition and need for care quickly. The crying happens there, and then you need to start doing something about this intimate violence situation…Like, I don’t have time for this, you need to talk about this with some other people on that side of things. (p.350)*

Another reason HCPs were reluctant to take responsibility for addressing IPA was the belief that their priority (or in some cases their sole purpose) should be to address medical complaints. For example, a psychiatrist in a study of mental health professionals’ responses to IPA explained:

*…Should we be addressing this [IPA]? Because I think so many things are coming under the role of psychiatry to sort out when actually they are not mental health problems. I suppose I struggle a bit with us taking on things that aren’t mental health problems…perhaps we should be directing people elsewhere* [46]*.(p.191)*

The argument that “social issues” such as IPA are not within the remit of a HCP was not unique to psychiatry or the mental health field, but spanned a range of professions and health settings. It was also present in studies conducted in many different countries. For example, general practitioners in a Greek study by Papadakaki and colleagues [41] saw their duty as a HCP as being limited to the treatment of injuries relating to IPA:

*A woman once came to my practice with a broken leg…she confessed that it was not an accident, but her husband lost control… I treated her wound, prescribed the necessary medication, explained to her how to care for the wound, and asked her to visit me again to monitor the healing process…this was my only duty as a doctor. (p.374)*

Similarly, in an Egyptian study [28], a surgeon stated that:

*I do not care if she fell down the stairs or her husband battered her, if she has a fracture I will fix it, that’s all I have to do. (p.97)*

Participants in a Finnish focus group study [38] argued that it was not appropriate or “natural” for a HCP to play the role of a friend and provide emotional support to women experiencing abuse.

*You can’t really hold the client’s hand and pat her head, it doesn’t really come somehow naturally. (p.351)*

Finally, there was a perception from some HCPs that the onus for disclosing IPA ought to be on the woman, rather than the practitioner [37, 47]. They felt that if a woman needed assistance, she should ask for it rather than waiting for the HCP to inquire. As a resident in a study in the ED by Sormanti and colleagues [47] argued: “Women are adults and should be able to bring up the issue [of IPA] themselves if they want help.” (p.33).

## Discussion

This qualitative meta-synthesis builds on previous reviews conducted by Sprague and colleagues [12] and Saletti-Cuesta et al. [13], examining the barriers to identification and response to IPA in health settings. However, where the existing evidence has explored barriers more broadly, we chose to focus specifically on the personal barriers that prevent HCPs from effectively addressing IPA in their daily practice. In doing this, we are not suggesting that systems-level issues such as a lack of time and resources are not critically important to overcome [21]. Rather, we wish to reinforce that many elements of what makes HCPs feel ready to respond to the challenging issue of IPA are related to the individual and their beliefs and attitudes [21]. These factors, although perhaps more difficult to identify than organisational barriers, in many ways could be easier to address through education, coaching and training programs [57, 58].

The first theme identified by our meta-synthesis – “*I can’t interfere”* – can be understood as a barrier to having a commitment. The belief that a HCP cannot get involved when they suspect a woman is experiencing IPA is one that has persistently been highlighted within the literature [12]. Across our included studies, HCPs expressed reluctance to intervene due to concern about damaging their relationship with the patient and because they viewed IPA as a private matter best left within the family. For a small number of HCPs working in high-conflict settings, there were also concerns for personal safety. These issues mirror those found by Sprague and colleagues [12], which is concerning to us given that 10 years have now passed. The findings also suggest that HCP perspectives are misaligned with what women actually want [17, 59]. In fact, studies consistently highlight that women are keen for HCPs to enquire about IPA and are unlikely to be offended as long as the practitioner asks in a sensitive and non-judgemental way [59]. We certainly do not disagree that identifying and responding to women experiencing IPA is difficult work that can take a personal toll on the HCP. Yet, Hegarty and McKibbin’s review on practitioner readiness [21] suggested that when HCPs are motivated by a broader ideological framework such as feminism, child rights or human rights, they may be more likely to have a commitment to addressing IPA, despite the challenges around raising the issue with their patients. Consequently, we strongly recommend that education and training programs for HCPs focus on identifying and reflecting on ideological belief systems that would facilitate a commitment to addressing IPA.

Another key finding of our review was the frustration and disillusionment HCPs felt when women went against their advice or repeatedly returned to the abusive partner (*“I don’t have control”*). Many HCPs saw their role as that of a “problem solver” who needed to “rescue” the patient; when they were unable to achieve this, they felt stressed, depressed and inadequate. There was also a disturbing need for some HCPs to be in control of women’s choices, feeling that they knew the best course of action. As far back as 1996, however, Gremillion and Kanof [60] argued that this fundamental misunderstanding of the HCP role could be a barrier to effective provision of care in the context of IPA. They suggested that, rather than seeking to take control, HCPs could “lessen their discomfort if they recognize that their role is as validator, listener, and advisor” (p.772). This certainly resonates with the findings of our recent meta-synthesis of women’s expectations of HCPs in the context of IPA [17]. We found that women wanted HCPs to facilitate choice and control in their interactions, and to provide advocacy and action that was guided by the woman’s individual needs and wishes [17]. Hegarty and McKibbin’s review [21] similarly identifies that adopting an advocacy approach rather than a controlling one is central to practitioner readiness to address IPA. However, an interesting point of difference is that Hegarty et al. found that papers discussing the need for practitioners to relinquish control of the clinical encounter were older, predating the shift towards patient-centered care. Our review includes only papers published since 2010, suggesting that – contrary to the finding of Hegarty and colleagues – the need to control women’s actions remains a barrier to some HCPs addressing IPA.

Lastly, we found that some practitioners were reluctant to accept the responsibility of addressing IPA with their female patients (*“I won’t take responsibility”*). For many, this reluctance was related to the perception of IPA as a “social problem” rather than a health issue, which – in their view – placed it outside the acceptable remit for a clinician. This is despite global attention being paid to the health consequences of IPA and the key role of health settings in addressing it [7, 61]. Furthermore, many HCPs across the included studies felt that providing emotional support to their female patients experiencing IPA was inappropriate or made them uncomfortable, citing that their role was simply to treat any physical injuries or refer them on to someone more qualified. Again, however, this is in opposition to what women expect from the clinical encounter. Qualitative research highlights the critical role of empathy, kindness and care in addressing IPA [17]. Contrary to the perceptions of HCPs in this review, demonstrating care can be done very simply, without any specific training (although evidence suggests that HCPs can also be trained in these skills if needed [62]). It is telling to consider the comment made by one participant in Finnbogadöttir et al.’s study: “*You can’t really hold the client’s hand and pat her head”* [35]*,* compared to this statement from a woman in a study by Reisenhofer and Sebold: “What I wanted was someone to sit on my bed and tell me that they understand, talk to me about some options that I may have had…and hold my hand” [63] (p2258). It is clear that some HCPs struggle to accept that health settings are an appropriate place to address IPA, or do not feel confident that they have the necessary skills to build up a trusting relationship with women experiencing violence. This is – in our view – an implementation problem rather than one stemming from a lack of awareness about the importance of person-centred or woman-centred care. The World Health Organization and other key bodies strongly advocate a woman-centred approach when responding to survivors of IPA [11], however, it is clear that translating the theory into practice is more challenging for HCPs than it might otherwise appear. This issue needs to be addressed in order to improve identification and response to IPA in health settings.

Our review did not identify many differences across professions or disciplines in the included studies; however, it was observed that nurses and midwives more often fell into the “I can’t interfere” category, being concerned about their relationship with the patient, whereas doctors and specialists tended to experience barriers that were more aligned with frustration, lack of control, and reluctance to take responsibility. This is consistent with the training each group of HCPs receives, with nurses more focused on holistic care [64] and doctors and specialists generally receiving training that is more biomedical [65]. Although some cultural nuances were observed within the dataset overall (e.g. a stronger reluctance from HCPS in some countries to interfere in “private matters”), the underlying sentiments were mostly consistent across the different countries.

**Fig. 3:**
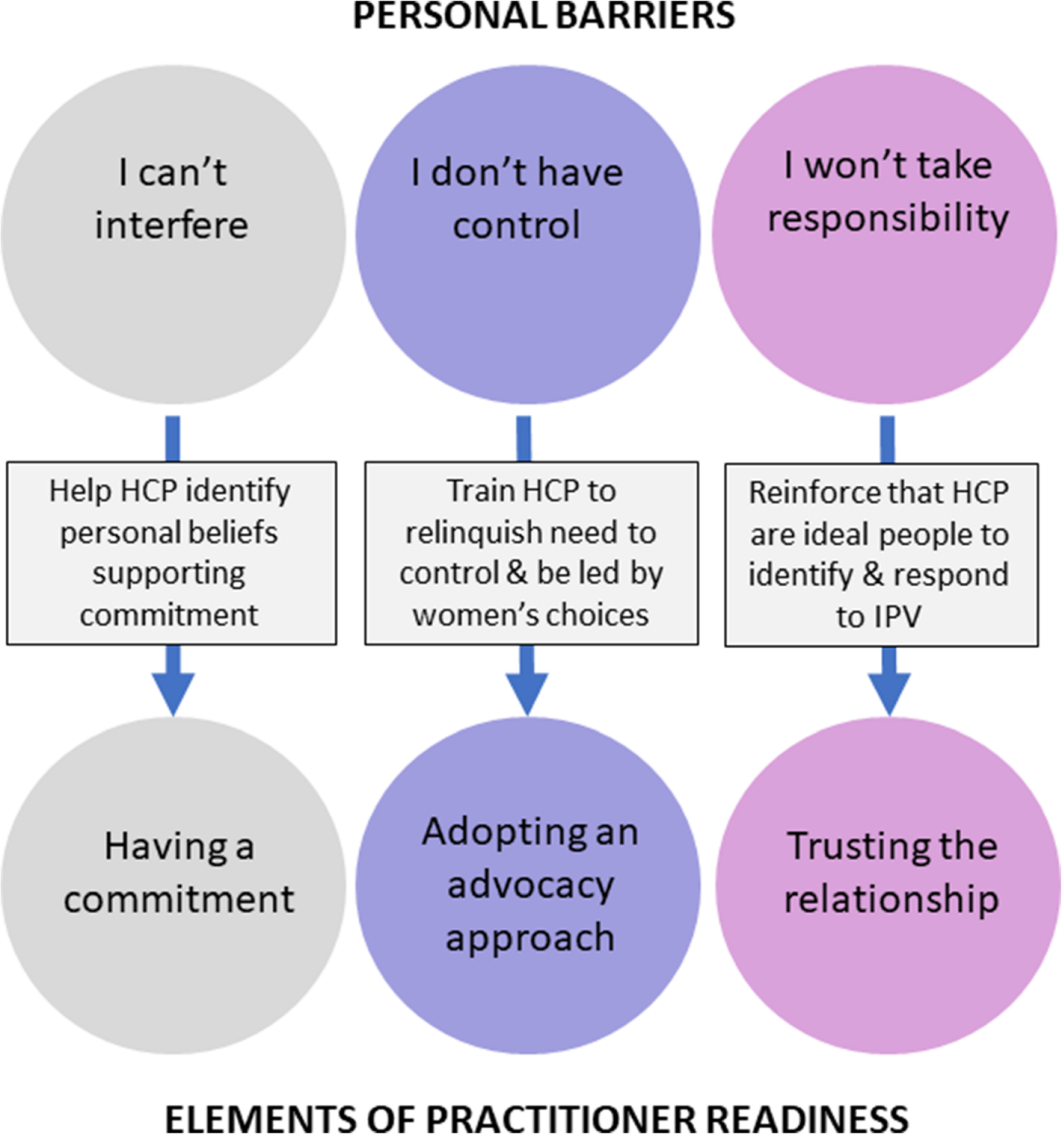
Addressing HCP-perceived barriers to responding to IPA

Our review has identified several key personal barriers that prevent HCPs from responding effectively to IPA in their daily practice. These barriers – lack of commitment, overemphasis on control, and reluctance to take responsibility – urgently need to be targeted in education and training programs. Figure 3 below suggests how each of these barriers could be addressed in order to shift HCPs towards a state of readiness to respond. The elements of readiness are drawn directly from the CATCH model developed in a previous review [21] and mentioned above.

### Strengths & limitations

A strength of this meta-synthesis is the diverse range of countries that were represented in this synthesis, as well as its multi-disciplinary team (encompassing both academics and clinician-researchers). A number of limitations also need to be acknowledged. First, although the CASP is considered to be a robust method of quality appraisal, it is not universally accepted that quality appraisal in qualitative studies is beneficial or meaningful [66, 67]. It is also debatable whether it identifies issues with study methodology, or with reporting [67]. Additionally, we did not assess the strength of the findings across the body of literature. Our findings should thus be interpreted with caution. Lastly, our review was limited to studies in English, although there were only a few studies excluded on this basis.

## Conclusions

Health practitioners experience a range of personal barriers to providing support to patients experiencing IPA, in addition to structural and organisational issues such as lack of time and workload pressure identified in previous reviews of the literature. The potential for personal barriers to be addressed through appropriate education, training and workplace support needs to be explored further. In particular, HCPs need to see themselves as having a critical role in improving the safety and wellbeing of women experiencing IPA but understand that this role involves supporting the woman in her individual choices. We suggest that this can be achieved by supporting HCPs to: identify and build upon underlying personal beliefs and value systems that motivate them to undertake the work of addressing IPA; develop strategies to manage frustration and assist with relinquishing control; and increase their trust and confidence that addressing IPA is within their capacity and skill-set. Future research ought to explore effective ways to do this within the context of a complex healthcare setting.

## Supplementary Information

**Additional file 1.**

## Data Availability

The datasets used and analysed during the current study are available from the corresponding author on reasonable request.
