# Supplementary material for: Personal barriers to addressing intimate partner abuse: a qualitative meta-synthesis of healthcare practitioners’ experiences
Source: BMC Health Serv Res. 2021 Jun 9;21:567. doi: 10.1186/s12913-021-06582-2 (PMC8191204; doi:10.1186/s12913-021-06582-2)
Supplement: Supplementary file 1 — Additional file 1. [file 12913_2021_6582_MOESM1_ESM.docx]

Search Strategy:

| **#** | **Searches** |
| --- | --- |
| 1 | Battered Women/ |
| 2 | (abuse$ adj3 wom#n).mp. |
| 3 | (abuse$ adj3 spous$).mp. [ |
| 4 | (abuse$ adj3 partner$).mp. |
| 5 | (abuse$ adj3 (wife or wives)).mp. [ |
| 6 | (batter$ adj3 (wife or wives)).mp. |
| 7 | (batter$ adj3 wom#n).mp. [ |
| 8 | (partner$ adj3 violen$).mp. |
| 9 | (spous$ adj3 violen$).mp |
| 10 | (gender adj3 violen$).mp. [ |
| 11 | domestic violence.mp. |
| 12 | family violence.mp |
| 13 | dating violence.mp. |
| 14 | marital rape.mp |
| 15 | reproductive coercion.mp. [ |
| 16 | Doctor*.mp. |
| 17 | nurse*.mp. |
| 18 | midwi*.mp. |
| 19 | dentist*.mp. |
| 20 | psychologist*.mp. |
| 21 | (health$ adj3 provider).mp. [ |
| 22 | (healthcare$ adj3 provider).mp. |
| 23 | (healthcare$ adj3 worker).mp. |
| 24 | (allied adj3 worker).mp. |
| 25 | health personnel.mp. or Health Personnel/ |
| 26 | Domestic Violence/ |
| 27 | Spouse Abuse/ |
| 28 | 1 or 2 or 3 or 4 or 5 or 6 or 7 or 8 or 9 or 10 or 11 or 12 or 13 or 14 or 15 or 26 or 27 |
| 29 | 16 or 17 or 18 or 19 or 20 or 21 or 22 or 23 or 24 or 25 |
| 30 | Qualitative Research/ |
| 31 | phenomenology.mp. |
| 32 | Hermeneutics/ |
| 33 | constructivism*.mp. |
| 34 | Interview/ |
| 35 | Social Sciences/ |
| 36 | theoretical orientation.mp. |
| 37 | questioning.mp. |
| 38 | information seeking.mp. |
| 39 | interviewing.mp. |
| 40 | Observation/ |
| 41 | Grounded Theory/ |
| 42 | Program Evaluation/ |
| 43 | verbal communication.mp. |
| 44 | Personal Narrative/ |
| 45 | disclosure analysis.mp. |
| 46 | content analysis.mp. |
| 47 | sociocultural factors.mp. |
| 48 | health attitudes.mp. |
| 49 | Attitude/ |
| 50 | client attitudes.mp. |
| 51 | consumer attitudes.mp. |
| 52 | female attitudes.mp. |
| 53 | community attitudes.mp. |
| 54 | cultural sensitivity.mp. |
| 55 | qualitative.mp. |
| 56 | ethno$.mp. |
| 57 | emic.mp. |
| 58 | etic.mp. |
| 59 | hermeneutic*.mp. |
| 60 | Heidegger*.mp. |
| 61 | husserl$.mp. |
| 62 | colaizzi$.mp. |
| 63 | giorgi$.mp. |
| 64 | glaser.mp. |
| 65 | strauss.mp. |
| 66 | van kaam$.mp. |
| 67 | van manen.mp. |
| 68 | constant compar$.mp. |
| 69 | (focus group$ or grounded theory or narrative analys$ or lived experience$ or life experience$ or theoretical sampl$ or purposive sampl$ or ricoeur or spiegelberg or merleau or metasynthes$ or meta-synthes$ or metasummar$ or meta-summar$ or metastud$ or meta-stud$ or maximum variation or snowball).mp. |
| 70 | (thematic$ adj3 analys$).mp. |
| 71 | (field note$ or fieldnote$ or field record$ or field stud$).mp. |
| 72 | (participant$ adj3 observ$).mp. |
| 73 | (nonparticipant$ adj3 observ$).mp. |
| 74 | (non-participant$ adj3 observ$).mp. |
| 75 | (semi-structured or semistructured or structured categor$ or unstructured categor$).mp. |
| 76 | (action research or audiorecord$ or taperecord$ or videorecord$ or videotap$).mp. [ |
| 77 | ((audio or tape or video$) adj5 record$).mp. |
| 78 | (interview$ or quasi-experiment$).mp |
| 79 | (case adj stud*).mp. |
| 80 | (collaborat* or consultat* or experience or involve* or narrative* or opinion* or participat* or partner* or perspective* or story or stories or view* or voice*).mp. |
| 81 | (self report or yarn*).mp. |
| 82 | Self Report/ |
| 83 | 30 or 31 or 32 or 33 or 34 or 35 or 36 or 37 or 38 or 39 or 40 or 41 or 42 or 43 or 44 or 45 or 46 or 47 or 48 or 49 or 50 or 51 or 52 or 53 or 54 or 55 or 56 or 57 or 58 or 59 or 60 or 61 or 62 or 63 or 64 or 65 or 66 or 67 or 68 or 69 or 70 or 71 or 72 or 73 or 74 or 75 or 76 or 77 or 78 or 79 or 80 or 81 or 82 |
| 84 | 28 and 29 and 83 |
